# Fimbria targeting superparamagnetic iron oxide nanoparticles enhance the antimicrobial and antibiofilm activity of ciprofloxacin against quinolone‐resistant *E. coli*

**DOI:** 10.1111/1751-7915.14327

**Published:** 2023-08-21

**Authors:** Nazli Atac, Kubra Onbasli, Irem Koc, Havva Yagci Acar, Fusun Can

**Affiliations:** ^1^ School of Medicine, Medical Microbiology Koç University Istanbul Turkey; ^2^ Koç University‐İşbank Center for Infectious Diseases (KUISCID) Istanbul Turkey; ^3^ Department of Metallurgical and Materials Engineering İstanbul Technical University Istanbul Turkey; ^4^ Graduate School of Materials Science and Engineering Koç University Istanbul Turkey; ^5^ Department of Chemistry Koç University Istanbul Turkey

## Abstract

High quinolone resistance of *Escherichia coli* limits the therapy options for urinary tract infection (UTI). In response to the urgent need for efficient treatment of multidrug‐resistant infections, we designed a fimbriae targeting superparamagnetic iron oxide nanoparticle (SPION) delivering ciprofloxacin to ciprofloxacin‐resistant *E. coli*. Bovine serum albumin (BSA) conjugated poly(acrylic acid) (PAA) coated SPIONs (BSA@PAA@SPION) were developed for encapsulation of ciprofloxacin and the nanoparticles were tagged with 4‐aminophenyl‐α‐D‐mannopyrannoside (mannoside, Man) to target *E. coli* fimbriae. Ciprofloxacin‐loaded mannoside tagged nanoparticles (Cip‐Man‐BSA@PAA@SPION) provided high antibacterial activity (97.1 and 97.5%, respectively) with a dose of 32 μg/mL ciprofloxacin against two ciprofloxacin‐resistant *E. coli* isolates. Furthermore, a strong biofilm inhibition (86.9% and 98.5%, respectively) was achieved in the isolates at a dose 16 and 8 times lower than the minimum biofilm eradication concentration (MBEC) of ciprofloxacin. Weaker growth inhibition was observed with untargeted nanoparticles, Cip‐BSA@PAA@SPIONs, confirming that targeting *E. coli* fimbria with mannoside‐tagged nanoparticles increases the ciprofloxacin efficiency to treat ciprofloxacin‐resistant *E. coli*. Enhanced killing activity against ciprofloxacin‐resistant *E. coli* planktonic cells and strong growth inhibition of their biofilms suggest that Cip‐Man‐BSA@PAA@SPION system might be an alternative and/or complementary therapeutic option for the treatment of quinolone‐resistant *E. coli* infections.

## Graphical abstract

Loading ciprofloxacin into SPIONs with further tagging the system to mannosides for fimbrial targeting have decreased the effective killing dose of ciprofloxacin by 4‐fold against two ciprofloxacin resistant *E.coli* isolates.

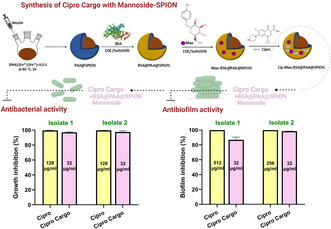

## INTRODUCTION

*Escherichia coli* is the most common bacteria isolated from urinary tract infections (Behzadi et al., 2020; Brannon et al., 2020). High prevalence of antibiotic resistance hardens the therapy's success (Can et al., 2015; Naziri et al., 2021). The most commonly prescribed antibiotic for uropathogenic *E. coli* treatment is ciprofloxacin, but high resistance rates to ciprofloxacin are seen among UTI *E. coli* (Stapleton et al., 2020). Biofilm formation of uropathogenic *E. coli* on surfaces and within intracellular host cells is also critical for recurrent infections (Flores‐Mireles et al., 2015; Manoharan et al., 2021).

Alternative approaches, such as nanoparticle‐based designs, are of interest for the treatment of antibiotic‐resistant *E. coli* infections as well as other bacteria types. Nanoparticles may act as antibacterial agents or drug‐delivery vehicles, offering an opportunity to overcome drug resistance and enhance the penetration of antibacterial drugs into biofilms (Gupta et al., 2019; Miller et al., 2015). Several nanoparticles, mainly silver and oxides of metals such as titanium and zinc have tendency to act as antimicrobial agents by producing free radical species and/or disrupting the cell integrity; however, their clinical adaptation is still limited (Goda et al., 2022; Miller et al., 2015; Shakerimoghaddam et al., 2017). Besides their antimicrobial activity, these particles can also trigger immune responses to defeat bacterial infections. Iron oxide nanoparticles, for instance, are of interest for their capacity to trigger macrophage polarization to enhance their generation of ROS and thus to eliminate bacterial growth both in planktonic and sessile level (Yu et al., 2020).

Superparamagnetic iron oxide nanoparticles (SPIONs) are usually accepted as biocompatible nanoparticles with several FDA‐approved compositions for magnetic resonance imaging and are also popular in magnetic transfection, magnetic hyperthermia, photothermal therapy and drug delivery (Arias et al., 2018; Chee et al., 2018; Chen et al., 2022). Indeed, there are several examples of the antibacterial activity of SPIONs. Leuba et al. reported a significant reduction in the growth of *S. aureus* biofilm depending on the surface functionality of SPIONs in favour of anionic ones: A 33.5, 31.1 and 28.1% decrease in bacterial growth was observed at 1 mg/mL SPION with carboxylate, isocyanate, amine functional SPIONs while only 18.7% reduction was seen with non‐functionalized SPIONs (Leuba et al., 2013). Javanbakht et al. also studied the influence of surface charge on the antibacterial properties of SPIONs and reported higher death of *Streptococcus mutans* (32%) with positively charged SPIONs than bare (25%) and negatively (19%) charged ones at 3 μg/mL SPION concentration (Javanbakht et al., 2016). In another study, anionic, poly(acrylic acid) (PAA) capped iron oxide nanoparticles prepared via a ligand exchange between oleate‐capped iron oxide nanoparticles and PAA penetrated into both gram‐negative *E. coli* and gram‐positive *S. aureus* and caused a significant inhibition of bacterial growth at concentrations over 30 mg iron/L (Nie et al., 2021).

Response of SPIONs to the external magnetic field was exploited to treat biofilms, as well. Taylor et al further conjugated SPIONs to iron, zinc or silver to obtain biofilm inhibition of *S. aureus* and obtained a 75% biofilm inhibition with zinc‐conjugated SPION (including 37 μg/mL Fe) (Taylor et al., 2012). Carboxymethyl chitosan‐coated magnetic iron oxide nanoparticles (CMCS‐MNPs) killed more than 99% *S. aureus* and *E. coli* planktonic cells at 2 mg/mL after 10 and 5 h incubation, respectively; however, there was no effect on biofilms. Penetration of the CMCS‐MNPs into the biofilms was achieved in the presence of an external magnetic field, and 84% and 95% reduction in cell viability of *S. aureus* and *E. coli* biofilms were achieved after 48 incubation (Chen et al., 2012). SPIONs may also deliver antibiotics: Polymersomes encapsulating both SPIONs and methicillin were developed to treat medical device‐associated infections utilizing the response of SPIONs to the magnetic field. Polymersomes penetrated into 20 μm thick *S. epidermis* biofilms under an external magnetic field and caused complete eradication of methicillin‐resistant biofilm at 40 μg/mL SPION and 20 μg/mL methicillin selectively, without damaging mammalian cells. Some nano‐formulations were successful even in the absence of a magnetic field: Co‐administration of PAA‐coated iron oxide nanoparticles and rifampicin provided 89% growth inhibition at 8 μg/mL rifampicin and 32 μg/mL SPION concentration by increasing the drug accumulation, while rifampicin showed only 42% growth inhibition at the same concentration (Padwal et al., 2014). Overall, these studies utilizing SPIONs indicate the need for high NP doses, dependence on surface functionalities and/or external magnetic field to deliver nanoparticles to biofilms for appreciable antibacterial effect from SPIONs. On the other hand, molecular targeting of SPIONs loaded with antibiotics has the potential for successful bacteria eradication by increasing the concentration of NPs on biofilm without a magnetic field at low NP and antibiotic doses (Geilich et al., 2017).

Bacterial binding to surfaces via fimbriae is the critical initiative step for biofilm formation (Sarkar et al., 2016) and might cause persisting infections due to colonization and production of a thick biofilm matrix. Hence, inhibition of the binding with mannosides has drawn significant attention (Cusumano et al., 2011; Mydock‐McGrane et al., 2016; Totsika et al., 2013). The fimbrial inhibition via drug conjugated organic/metallic nanoparticles (Sanchez et al., 2021) emerged as a promising treatment option. Currently, the antibacterial effects of various nanoparticles are being examined using various designs, including conjugation to existing antibiotics (Grumezescu et al., 2014; Wang et al., 2017) for localized and efficient treatments as well as the fimbrial binding inhibition to prevent biofilm formation (Mousavifar et al., 2019).

Herein, we aim to improve antibacterial and antibiofilm activity on antibiotic‐resistant uropathogenic *E. coli* via drug‐loaded nanoparticles targeting fimbriae via mannoside tags, inhibiting fimbrial binding and enhancing drug delivery. Specifically, ciprofloxacin‐loaded SPION tagged with mannoside is suggested for the treatment of ciprofloxacin‐resistant *E. coli*. PAA‐coated SPIONs, confirmed as biocompatible in our previous studies, were prepared and then conjugated with bovine serum albumin (BSA) for loading ciprofloxacin. BSA is very effective in the entrapment of hydrophobic drugs and offers biocompatibility, stability, biodegradability and low immunogenicity. 4‐Aminophenyl‐α‐d‐mannopyrannoside was conjugated to BSA@PAA@SPIONs loaded with ciprofloxacin to target *E. coli* fimbriae. With this nanoparticle composition (Cip‐Man‐BSA@PAA@SPION), we achieved a strong bactericidal activity, particularly in biofilms of the two ciprofloxacin‐resistant *E. coli* isolates with an 8–16 times reduction of the effective ciprofloxacin dose from 256 and 512 μg/mL to 32 μg/mL, respectively.

## METHODS

### Materials

Iron (II) chloride and iron (III) chloride were purchased from Merck. Poly (acrylic acid) was obtained from Sigma. Bovine serum albumin was provided by Gen‐Depot. Ethyl‐3‐(3‐dimethylaminopropoyl) carbodiimide (EDC) and N‐hydroxysulfosuccinimide (sulfo‐NHS) were purchased from Thermo Scientific. 4‐Aminophenyl α‐d‐mannopyranoside and ciprofloxacin were supplied from Sigma‐Aldrich.

### Iron oxide nanoparticle synthesis

Poly (acrylic acid) (PAA) coated SPIONs were synthesized by a co‐precipitation method using FeCl_3_.6H_2_O and FeCl_2_.4H_2_O under an argon atmosphere as described in detail before (Bilici et al., 2018, 2020). Briefly, iron salts ([Fe^3+^]: [Fe^2+^] = 2:1) and PAA were dissolved in deoxygenated water for 20 min at 85°C and then NH_4_OH was added into the solution. After 1 h reaction, the solution was cooled to room temperature and placed on a magnet overnight to remove any precipitated particles. Finally, PAA@SPIONs were washed several times with DI water using centrifugal filters (5 kDa centrifugal filters) and stored at room temperature.

### BSA conjugation to PAA@SPIONs

BSA was conjugated to PAA@SPIONs via amidation reaction as described previously (Bilici et al., 2020): PAA@SPIONs (25.6 mg/mL, 7 mL) were activated with 20.1 mg EDC and 22.8 mg sulfo‐NHS in MES buffer at pH 6 for 30 min. Then, the MES buffer was replaced with PBS (pH = 7.4) using centrifugal filters (5 kDa cut‐off). Then, 1.16 gr BSA in PBS (5 mL) was added to this solution and stirred at +4°C for 48 h. The reaction mixture was quenched with hydroxylamine and washed with PBS through 100 kDa centrifugal filters. Bradford assay was used to determine the amount of BSA conjugated to SPIONs. Briefly, BSA at different concentrations was used to prepare a calibration curve. A sample of washed BSA conjugated SPIONs and 1 mL of BSA references at different concentrations were mixed with 1 mL of Bradford agent and shaken for 10 min at room temperature. Then, the absorbance of each was determined at 595 nm using UV–Vis spectroscopy. The amount of unconjugated BSA was determined from the calibration curve and subtracted from the initial amount to calculate the bound BSA amount. The efficiency of BSA conjugation to SPIONs was determined as 84.8%.

### Mannoside conjugation to BSA@PAA@SPIONs

BSA@PAA@SPIONs were activated with EDC/sulfoNHS in MES using 2.2698 × 10^−5^ mole of EDC and sulfo‐NHS. After buffer exchange with PBS, 4‐aminophenyl α‐d‐mannopyranoside (Mannoside) (3.783 × 10^−6^ mole) was added to SPIONs in PBS. After 24 h mixing at room temperature, the reaction was quenched with hydoxylamine and nanoparticles was washed with PBS through centrifugal filters (5 kDa centrifugal filters). The mannoside concentration on SPIONs was calculated using a phenol‐sulfuric acid colorimetric assay. A calibration curve was prepared with different concentrations of mannoside. Briefly, 0.5 mL phenol solution (5%) and 2.5 mL sulfuric acid were added to 0.5 mL of nanoparticles and different concentrations of mannoside. The solutions were kept at room temperature for 10 min and then put in water bath for 20 min. The absorbance of these solutions was recorded using UV–Vis spectroscopy at 486 nm. The amount of unconjugated mannoside was calculated using the calibration curve and subtracted from the initial loading for the determination of the bound mannoside amount.

### Ciprofloxacin loading to nanoparticles

Ciprofloxacin (5 mg/mL) was added dropwise to BSA@PAA@SPION and Man‐BSA@PAA@SPION (2.5 mg/mL, 10 mL) at pH 4–5 and mixed overnight at room temperature. The electrostatic loading of ciprofloxacin to SPIONs at acidic pH was confirmed by isothermal titration calorimetry (Affinity ITC, USA). Observed strong binding exotherms in Figure S1 confirmed the successful binding of ciprofloxacin to BSA@PAA@SPION.

### Characterization

The nanoparticles' hydrodynamic sizes and zeta potential were measured using a Malvern Zetasizer Nano ZS. The iron content of nanoparticles was determined by an Agilent 7700XICP‐MS inductively coupled plasma‐mass spectrometer after the nanoparticles were digested using acid mixture (ultrapure H_2_SO_4_ and HNO_3_ [1:9 v/v]).

### Bacterial isolates

Two urinary tract infection *E. coli* isolates (Isolate no 230 and 294) resistant to ciprofloxacin were used in the experiments. Minimum Inhibitory Concentrations (MIC) were determined with broth micro‐dilution as 128 μg/mL (CLSI, Clinical and Laboratory Standards Institute, 2010). Viable bacterial counts were recorded to define Minimum Bactericidal Concentrations (MBC) also as 128 μg/mL for both isolates (Passerini de Rossi et al., 2009).

### Antibacterial activity of the agents on planktonic bacteria

A single colony of the isolates was inoculated in Tryptic Soy Broth (TSB, BD™ Bacto™) at 37°C at 135 rpm for 10 h. Then, bacterial concentration was set to 10^6^ CFU/mL and incubated for 24 h with BSA@PAA@SPION, BSA@PAA@SPION‐Ciprofloxacin (abbreviated as Cip‐BSA@PAA@SPION), BSA@PAA@SPION‐4‐Aminophenyl‐α‐D‐mannopyrannoside (abbreviated as Man‐BSA@PAA@SPION) and BSA@PAA@SPION‐ 4‐Aminophenyl‐α‐d‐mannopyrannoside‐Ciprofloxacin (abbreviated as Cip‐Man‐BSA@PAA@SPION) with a final concentration of 32 μg/mL ciprofloxacin (corresponding to 52 μg/mL Fe). The control culture was incubated with TSB. Viable cells were determined by the plate spread method to count the colonies after serial ten‐fold dilutions. All experiments were done in triplicate. Growth reduction of >90.0% was considered a strong inhibition (Balouiri et al., 2016; Pankey & Sabath, 2004).

### Multiphoton microscopy imaging

Planktonic cultures were stained with BacLight Live‐Dead Bacterial Viability kit (Invitrogen, L7007) for 1 h at dark. Fluorescence images were recorded via multiphoton microscope (Leica TCS SP8 MP) using 25x water objective. Excitation and emission wavelengths were 480/500 nm and 490/635 nm.

### Anti‐biofilm activity of the agents

Single colony inoculation was performed similarly to the steps described above, and after the overnight incubation, 100 μL of bacterial suspension was transferred to low salt Lennox LB (BD™ Bacto™) with a bacterial concentration of 10^7^ CFU/mL. After 8 h, 100 μL of fresh Lennox LB was added to wells to induce fimbrial attachment before biofilm formation (Totsika et al., 2011). At the end of the second FimH induction, the medium was removed, and fresh M9 minimal medium (BD™ Bacto™) was added to wells and incubated at 37°C for 24 h. Biofilm production of the isolates was compared with crystal violet staining. Mean optical density (OD) values were measured at 540 nm (Merritt et al., 2005) and OD >0.5 was considered a thick biofilm producer. The minimum biofilm eradication concentration (MBEC) of ciprofloxacin was determined by MBEC assay by incubation of biofilms with 128, 256, 1024 and 2048 μg/mL ciprofloxacin concentrations overnight (Passerini de Rossi et al., 2009). Ciprofloxacin dose that inhibited the 99.9% of the biofilm is considered to be MBEC.

In order to analyse the effect of agents on *E. coli* biofilms, overnight incubation of biofilms with BSA@PAA@SPION, Cip‐BSA@PAA@SPION, Man‐BSA@PAA@SPION and Cip‐Man‐BSA@PAA@SPION with a final concentration of 32 μg/mL ciprofloxacin (Corresponding to 52 μg/mL Fe) was performed. Fresh M9 medium was added to control wells. The colony counts of the isolates within biofilms were determined as described above following 5 min of plate sonication at 75 Hz. All experiments were done in triplicate.

### Statistical analysis

Viable bacterial counts were compared with a one‐way ANOVA test using GraphPad Prism 9. Inhibition caused by BSA@PAA@SPION, Man‐BSA@PAA@SPION, Cip‐BSA@PAA@SPION and Cip‐Man‐BSA@PAA@SPION were compared to control viable counts. Inhibition was considered as statistically significant for *p* < 0.05.

## RESULTS and DISCUSSION

### Preparation and characterization of Mannoside conjugated ciprofloxacin loaded SPIONs

**FIGURE 1:**
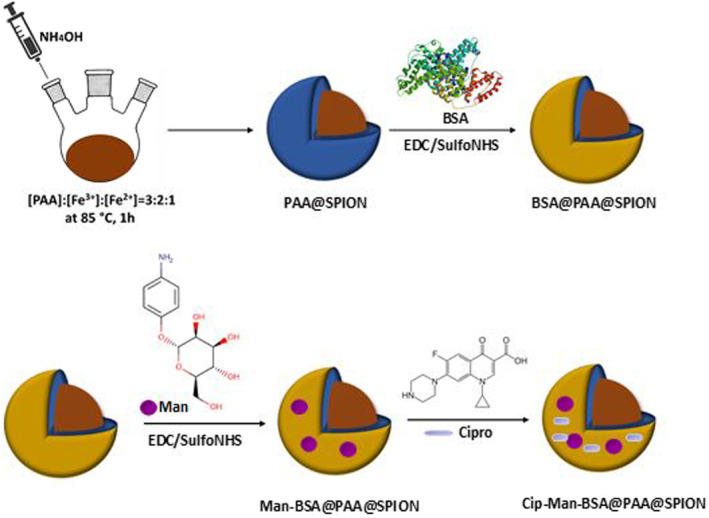
Synthesis of mannoside‐tagged and ciprofloxacin (Cip) loaded BSA@PAA@SPIONs.

The mannoside‐tagged and ciprofloxacin‐loaded BSA@PAA@SPIONs (Figure 1) were developed to treat ciprofloxacin‐resistant uropathogenic *E. coli* infections. Anionic, PAA‐coated SPIONs were synthesized using the co‐precipitation method based on our previous studies. For the loading of hydrophobic ciprofloxacin, nanoparticles were conjugated with bovine serum albumin (BSA) via an amide bond. 4‐Aminophenyl α‐d‐mannopyranoside was conjugated to NPs to improve their uptake via recognition of NPs by fimbriae of uropathogenic *E. coli*. This conjugation was accomplished with 51.33% efficiency based on phenol‐sulfuric acid colorimetric assay. As a final step, ciprofloxacin was loaded to both BSA@PAA@SPION and Man‐BSA@PAA@SPION, producing the Cip‐Man‐BSA@PAA@SPION with a final concentration of 32 μg ciprofloxacin/ml and 52 μg Fe/ml. Efforts to produce nanoparticles with higher ciprofloxacin content failed due to particle instability at higher drug loading resulting in particle precipitation. Therefore, all further investigations were made with this final composition.

Table 1 shows the hydrodynamic sizes and surface charges of prepared NPs in this study. BSA@PAA@SPIONs have a number‐based hydrodynamic size of 4.49 nm and a ζ potential of −35.5 mV, confirming their anionic nature. Conjugation of mannoside to BSA@PAA@SPIONs did not cause a significant change in hydrodynamic size (4.9 nm) or surface charge (−33 mV). On the other hand, ciprofloxacin loading to Man‐BSA@PAA@SPIONs slightly increased the hydrodynamic size to 41.19 nm and reduced the surface charge to −22 mV. Overall, the final nanoparticle composition was achieved in small size with negative surface charge.

**TABLE 1:** Hydrodynamic sizes and surface charges of prepared NPs.

Samples	Number‐based hydrodynamic size (nm)	PDI	Surface charge (mV)
BSA@PAA@SPION	4.49 ± 1.5	0.523	−35.5 ± 3.8
Man‐BSA@PAA@SPION	4.9 ± 1.6	0.587	−33 ± 5.1
Cip‐Man‐BSA@PAA@SPION	41.19 ± 1.5	0.433	−22 ± 2.8
Cip‐BSA@PAA@SPION	7.363 ± 1.3	0.427	−32.5 ± 3.3

### Antibacterial activity of the agents on planktonic bacteria

Treatment of quinolone‐resistant *E. coli* infections is challenging due to the bacterium's strong interaction with the host epithelial cells through specific fimbriae and the potent nature of multidrug resistance (MDR) (Bunduki et al., 2021). Therefore, inhibition of fimbrial binding and/or using targeted nanoparticle‐based delivery systems for enhancing antimicrobial activity are critical strategies for overcoming the limitations of antibiotic treatment (Qindeel et al., 2021). For this purpose, we designed BSA@PAA@SPION tagged with 4‐aminophenyl‐α‐D‐mannopyrannoside to target *E. coli* fimbriae and by ciprofloxacin loading to these nanoparticles, we aimed to increase the ciprofloxacin treatment efficiency on ciprofloxacin‐resistant UTI *E. coli* isolates.

**FIGURE 2:**
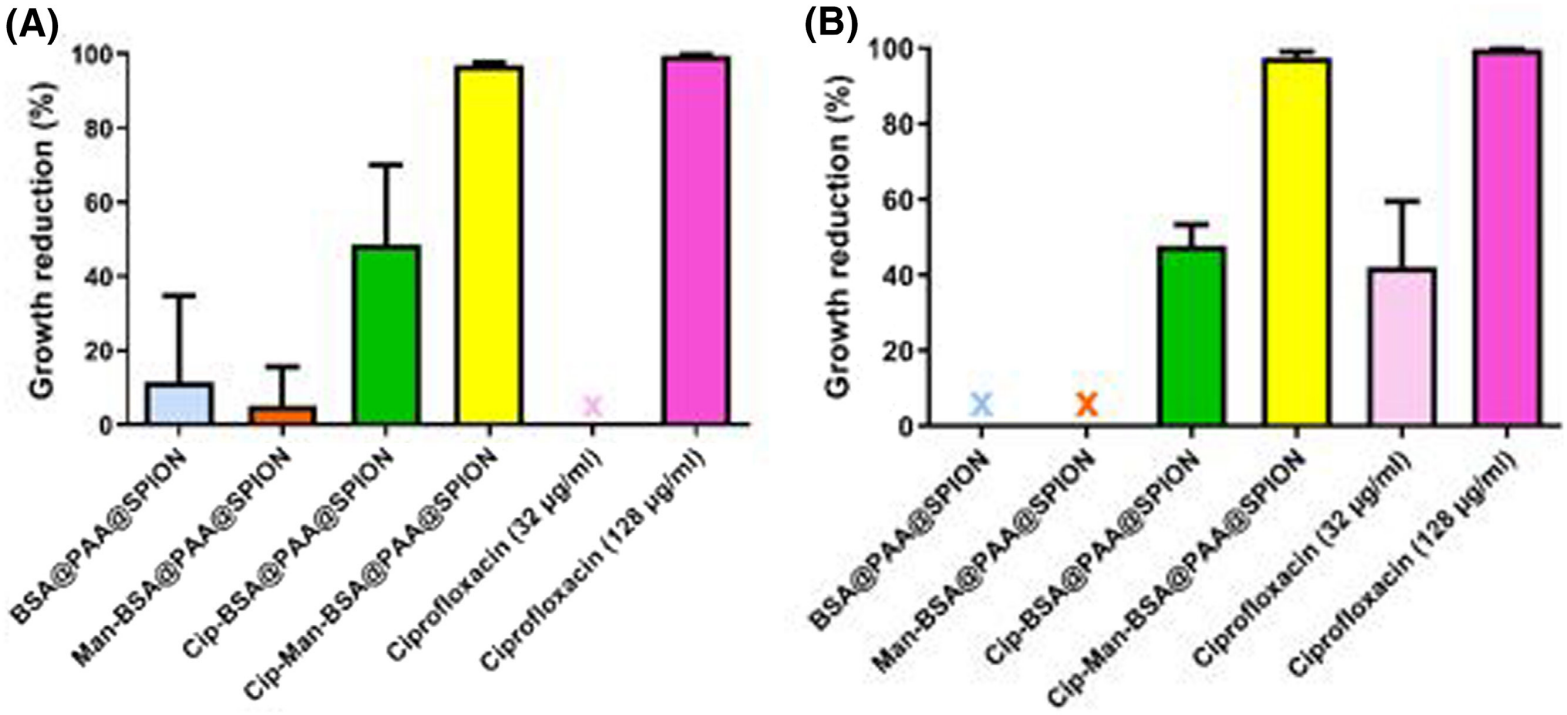
Growth reduction (%) in planktonic cells of *E. coli* isolates: A) Isolate 230; B) Isolate 294. Ciprofloxacin content of nanoparticles: 32 μg/mL.

Antibacterial activity of BSA@PAA@SPION, Cip‐BSA@PAA@SPION, Man‐BSA@PAA@SPION and Cip‐Man‐BSA@PAA@SPION on planktonic cells were tested at 32 μg/mL and 128 μg/mL ciprofloxacin doses (Figure 2). Growth reduction with Cip‐Man‐BSA@PAA@SPION was found to be 97.1% for isolate 230 (2.41 × 10^8^ CFU/mL & *p*: 0.0097) and 97.5% for isolate 294 (1.16 × 10^8^ CFU/mL & *p*: 0.0118) at 32 μg/mL ciprofloxacin. Without mannoside tagging, the growth reduction with Cip‐BSA@PAA@SPION was 48.5% (Mean CFU/mL 4.22 × 10^9^) and 47.6% (Mean CFU/mL 2.45 × 10^9^) for isolates 230 and 294, respectively. Ciprofloxacin alone at 32 μg/mL concentration did not cause a strong inhibition (no inhibition for 230 and 42.2% inhibition for 294), whereas the MBC doses of isolates (128 μg/mL) killed 99.99% of the isolates (3.33 × 10^7^ and 1.40 × 10^7^ for isolates 230 and 294). The mean CFU/mL values for control were 8.20 × 10^9^ and 4.07 × 10^9^, respectively. No growth reduction (<15%) was observed either with BSA@PAA@SPION or Man‐BSA@PAA@SPION.

These results indicate the success of targeted delivery of the drug via mannoside‐tagged nanoparticles. By this delivery system, we achieved fourfold decrease (from 128 to 32 μg/mL) in the effective killing concentration of ciprofloxacin.

**FIGURE 3:**
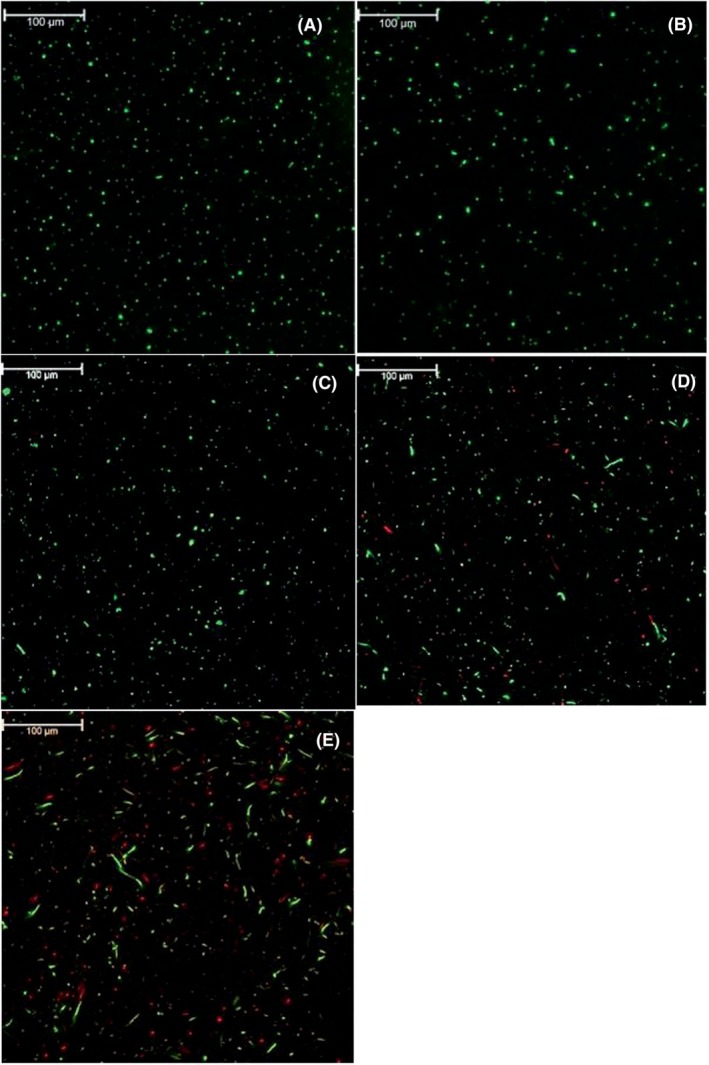
Confocal microscope images of isolate 294: A) Bacterial control; B) BSA@PAA@SPION, C) Man‐BSA@PAA@SPION, D) Cip‐BSA@PAA@SPION and E) Cip‐Man‐BSA@PAA@SPION treated bacteria. Ciprofloxacin content of nanoparticles: 32 μg/mL. Red: Dead bacteria, Green: Live bacteria. Images were taken with 25x water objective.

The killing activity of the agents on the planktonic cells was confirmed via live/dead cell assay on the treated planktonic culture of isolate 294 under a confocal microscope (Figure 3). In agreement with the growth inhibition data, Cip‐Man‐BSA@PAA@SPION provided the highest amount of dead cells and also caused elongation of the bacteria (Figure 3E), which is a sign of disrupted morphology. This was observed with Cip‐BSA@PAA@SPION as well (Figure 3D), but to a lower extent. No dead cells or morphology change was observed when bacteria were treated with BSA@PAA@SPION (Figure 3B) or Man‐BSA@PAA@SPION (Figure 3C). Hence, nanoparticles are safe, but they deliver the toxic drug to the bacterial cells, especially when tagged with mannoside, causing significant toxicity in the ciprofloxacin‐resistant *E. coli*. Javanbakhkt et al. compared the effect of surface charge of SPIONs on their bactericidial activity and showed lower antibacterial activity (higher abundance of viable cells) with negatively charged SPIONs (1–3 μg/mL) of against *Streptococcus mutans* biofilm (Javanbakht et al., 2016) with a similar trend in planktonic culture as well. Our results coincide with this study that negatively charged BSA@PAA@SPION alone (Figure 3B) did not cause bacterial inhibition and viable cell ratio (green) are similar to that of control (Figure 3A). Similarly, Arakha et al. compared the surface charge of iron oxide nanoparticles and obtained high antibacterial activity with 50 μM positively charged iron oxide nanoparticles against *E. coli* (Arakha et al., 2015).

Nie et al. reported 99.99% bacterial inhibition in *E. coli* and *S. aureus* with 30 and 60 μg/mL dose of PAA‐capped iron oxide nanoparticles (Nie et al., 2021). However, we did not detect an antibacterial activity with BSA@PAA@SPION alone, with 52 μg/mL iron dose. We suggest that hydrodynamic size, surface charge and coating properties are the critical factors that can cause such differences in the results (Gudkov et al., 2021).

Likewise, the previous studies reported the increased efficiency of ciprofloxacin after conjugation with silver or gold nanoparticles (Kooti et al., 2018; Sreedharan & Singh, 2019). Kooti et al. showed the enhanced antibacterial activity of ciprofloxacin against *E. coli* ATCC® 25922™ when encapsulated in Graphene oxide‐Cobalt ferrite‐Silver nanocomposite (Kooti et al., 2018). Sreedharan et al. also reported increased antibacterial activity with ciprofloxacin conjugated to gold nanoflowers (Sreedharan & Singh, 2019). These studies, however, did not include the antibiofilm activity of these nanoparticle systems and the nanoparticles lacked bacterial targeting.

### Antibiofilm activity of the agents

**FIGURE 4:**
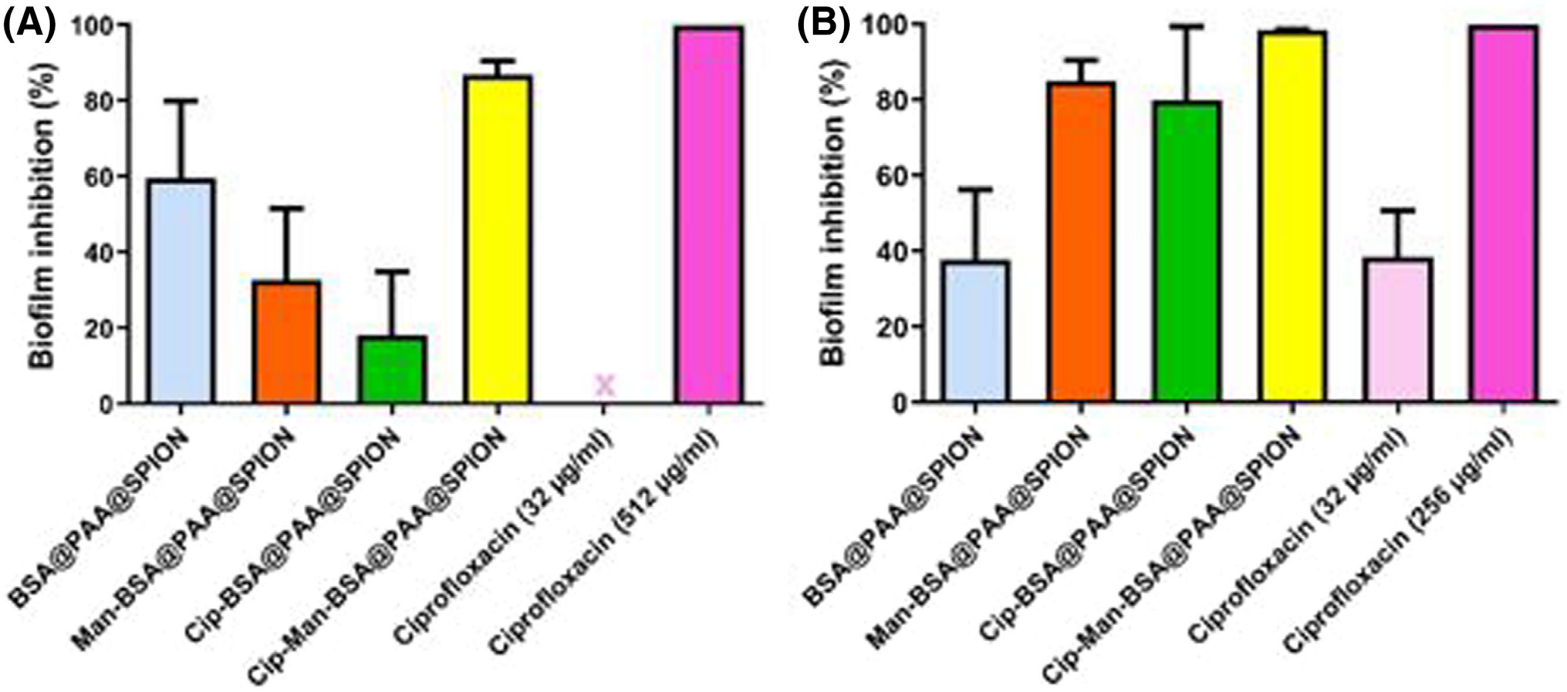
Biofilm inhibition (%) of *E. coli* isolates: A) Isolate 230 (Thick biofilm producer); B) Isolate 294 (Thin biofilm producer). Ciprofloxacin content of BSA@PAA@SPIONs: 32 μg/mL.

Biofilms of isolates 230 and 294 were developed and confirmed with crystal violet staining. Isolate 230 produced thicker biofilm than 294 with mean OD values (540 nm) of 0.665 and 0.354, respectively. MBEC of ciprofloxacin for isolate 230 and 294 were 512 and 216 μg/mL, respectively. Biofilms were treated with nanoparticles and free ciprofloxacin. All agents caused weaker biofilm inhibition for isolate 230, producing thicker biofilm than isolate 294 (Figure 4). This difference might be due to the poorer penetration of the delivery system into the thick biofilm matrix.

While free ciprofloxacin inhibited the biofilm of isolate 230 at 512 μg/mL, Cip‐Man‐BSA@PAA@SPION with 32 μg/mL ciprofloxacin content caused 86.9% inhibition (2.30 × 10^9^ CFU/mL & *p*: 0.0486) in the biofilm. On the other hand, Cip‐BSA@PAA@SPION caused 18.1% (1.23 × 10^10^ CFU/mL) and Man‐BSA@PAA@SPION caused 32.7% reduction (1.18 × 10^10^ CFU/mL) in biofilm mass. Colony count of control was 1.75 × 10^10^ for isolate 230. Biofilm inhibition rates with 32 μg/mL Cip‐Man‐BSA@PAA@SPION were 98.5% (4.30 × 10^8^ CFU/mL & *p* < 0.0001) and 79.9% (5.75 × 10^9^ CFU/mL) with Cip‐BSA@PAA@SPION for weak biofilm producer isolate 294. Without ciprofloxacin, Man‐BSA@PAA@SPION caused 85.0% (4.30 × 10^9^ CFU/mL) inhibition in biofilm mass of isolate 294 suggesting the potent interaction of mannoside molecules with polysaccharides causing inhibition of further thickening of the biofilm (Colony count of control: 2.86 × 10^10^).

The lack of growth reduction in planktonic cells but a high reduction of biofilm mass with ciprofloxacin lacking Man‐BSA@PAA@SPION strongly suggests that mannoside does not affect cellular components of the biofilm but the extracellular polysaccharide matrix. Fimbrial attachment is critical for biofilm growth, where mannoside binding to the surface of fimbriae disrupts the biofilm mass, causing loss of function (Mydock‐McGrane et al., 2016). Zhu et al. showed a strong correlation between the binding affinities of immobilized mannoside derivatives and the early‐stage adhesion and biofilm formation of fim + *E. coli* 83,972 (Zhu et al., 2020). Penetration and interaction of the mannoside‐tagged nanoparticles would be easier within thinner biofilms, explaining the stronger inhibition of the thinner biofilm producer (isolate 294) with mannoside‐tagged nanoparticles.

The weak anti‐biofilm activity of Cip‐BSA@PAA@SPION in the thick biofilm (18.1%) is due to limited penetration of the drug into biofilm (Rafaque et al., 2020). In our study, MBEC of ciprofloxacin for thick biofilm producer isolate 230 and thin biofilm producer isolate 294 were 512 and 256 μg/mL, respectively. The previous studies reported that up to 10 × MIC of ciprofloxacin should be administered to *E. coli* biofilms for 99.99% inhibition and 64 × MIC required for eradication of *P. aeruginosa* biofilms (Macia et al., 2014; Rodriguez‐Martinez et al., 2007). Such an increase in MBEC doses causes treatment failures. However, we achieved a strong antibiofilm activity with 32 μg/mL ciprofloxacin being 16 and 8 times lower than the corresponding MBECs, when conjugated to Man‐BSA@PAA@SPION. With this in consideration, our results indicate the success of targeted delivery of the ciprofloxacin into *E. coli* biofilm mass.

There are other efforts to inhibit the attachment of *E. coli* to urinary tract, either with oral mannosides (Han et al., 2012) or, for example, with N‐Acetylcysteine (NAC). Manoharan et al. used 4.89 mg/mL N‐Acetylcysteine (NAC) to decrease *E. coli* biofilm with 5‐log inhibition (Manoharan et al., 2021). The use of nanoparticles is now important as alternative therapy approaches for bacterial inhibition to defeat the limitations due to antibiotic resistance (Alabresm et al., 2020; Batul et al., 2020). However, our study is unique in combining BSA@PAA@SPIONs and 4‐aminophenyl‐α‐D‐mannopyrannoside to target *E. coli* fimbriae and design an efficient ciprofloxacin delivery system for ciprofloxacin‐resistant *E. coli* isolates.

## CONCLUSION

We have achieved a dramatic killing activity on ciprofloxacin‐resistant *E. coli* via enhanced drug delivery and strong growth inhibition of their biofilms by targeted inhibition of polysaccharide production in the matrix with the use of Cip‐Man‐BSA@PAA@SPION drug delivery system. Cip‐Man‐BSA@PAA@SPION system, and the approach represented by this nanoparticle composition, might be an alternative and/or complementary therapy option for the treatment of quinolone‐resistant *E. coli* infections in the era of nano‐antibiotic therapies.

## AUTHOR CONTRIBUTIONS

**Nazli Atac:** Conceptualization (equal); data curation (equal); formal analysis (equal); funding acquisition (equal); investigation (equal); methodology (equal); project administration (equal); resources (equal); software (equal); supervision (equal); validation (equal); visualization (equal); writing – original draft (equal); writing – review and editing (equal). **Kubra Onbasli:** Conceptualization (equal); data curation (equal); formal analysis (equal); funding acquisition (equal); investigation (equal); methodology (equal); project administration (equal); resources (equal); software (equal); supervision (equal); validation (equal); visualization (equal); writing – original draft (equal); writing – review and editing (equal). **Irem Koc:** Conceptualization (equal); data curation (equal); formal analysis (equal); funding acquisition (equal); investigation (equal); methodology (equal); project administration (equal); resources (equal); software (equal); supervision (equal); validation (equal); visualization (equal); writing – original draft (equal); writing – review and editing (equal). **Havva Yagci Acar:** Conceptualization (equal); data curation (equal); formal analysis (equal); funding acquisition (equal); investigation (equal); methodology (equal); project administration (equal); resources (equal); software (equal); supervision (equal); validation (equal); visualization (equal); writing – original draft (equal); writing – review and editing (equal). **Fusun Can:** Conceptualization (equal); data curation (equal); formal analysis (equal); funding acquisition (equal); investigation (equal); methodology (equal); project administration (equal); resources (equal); software (equal); supervision (equal); validation (equal); visualization (equal); writing – original draft (equal); writing – review and editing (equal).

## CONFLICT OF INTEREST STATEMENT

The authors declare no conflicts of interest.

## Supporting information


Figure S1.
