# Supplementary material for: Fimbria targeting superparamagnetic iron oxide nanoparticles enhance the antimicrobial and antibiofilm activity of ciprofloxacin against quinolone‐resistant E. coli
Source: Microb Biotechnol. 2023 Aug 21;16(11):2072–81. doi: 10.1111/1751-7915.14327 (PMC10616650; doi:10.1111/1751-7915.14327)
Supplement: Supplementary file 1 — Figure S1. [file MBT2-16-2072-s001.docx]

**Fimbria targeting Superparamagnetic Iron Oxide Nanoparticles enhance the antimicrobial and antibiofilm activity of ciprofloxacin against quinolone-resistant *E.coli***

Nazli Atac^ᵻa,b^, Kubra Onbasli^ᵻc^, Irem Koc^d^, Havva Yagci Acar^d,e*^ and Fusun Can^a,b*^

^a^ Koç University, School of Medicine, Medical Microbiology, Rumelifeneri Yolu,

Sarıyer, İstanbul, Türkiye.

^b^ Koç University-İşbank Center for Infectious Diseases (KUISCID), Topkapı,

İstanbul, Türkiye.

^c^ İstanbul Technical University, Department of Metallurgical and Materials Engineering, Ayazağa, İstanbul, Türkiye.

^d^ Koç University, Graduate School of Materials Science and Engineering,

Rumelifeneri Yolu, Sarıyer, İstanbul, Türkiye.

^e^ Koç University, Department of Chemistry, Rumelifeneri Yolu, Sarıyer, İstanbul,

Türkiye.

ᵻ Co-first author

*Co-corresponding author


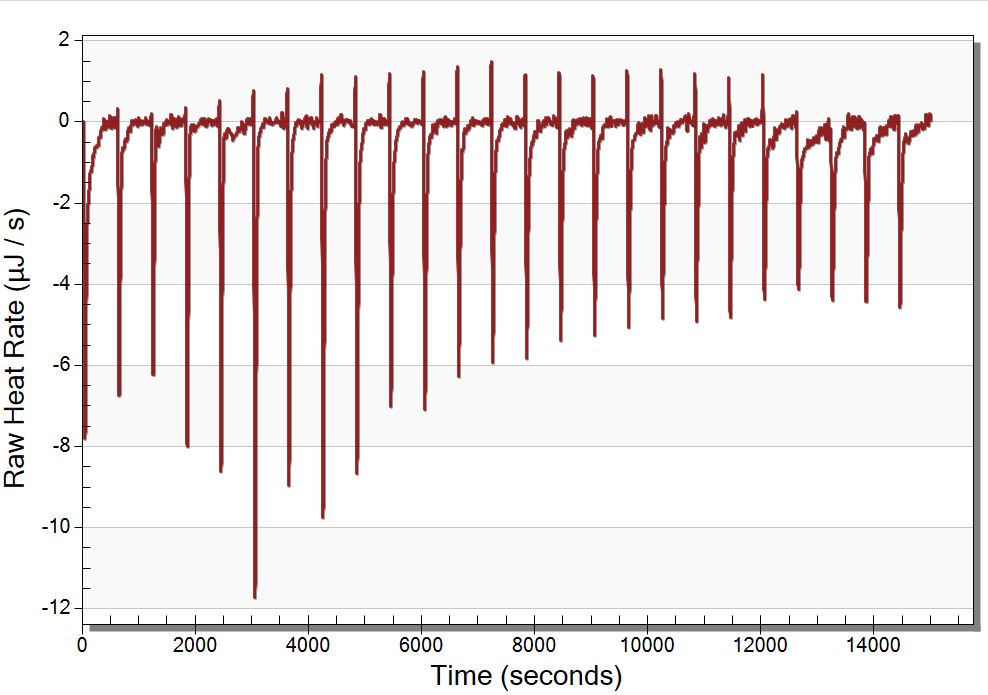


**Figure S1.** Binding exotherms of ciprofloxacin to BSA@PAA@SPION detected via isothermal titration calorimetry (ITC).
